# Impact of Pancreatic Stump Wrapping with Mesh on Post-Operative Pancreatic Fistula in Patients Undergoing Distal/Left Pancreatectomy for Malignant or Benign Diseases: A Systematic Review and Meta-Analysis

**DOI:** 10.3390/medicina61091688

**Published:** 2025-09-17

**Authors:** Andrea Morini, Maurizio Zizzo, Magda Zanelli, Lorenzo Dell’Atti, Federica Mereu, Andrea Palicelli, Mario Giuffrida, Elena Orlandi, Massimiliano Fabozzi

**Affiliations:** 1 Surgical Oncology Unit, Azienda USL—IRCCS di Reggio Emilia, 42123 Reggio Emilia, Italy; maurizio.zizzo@ausl.re.it (M.Z.); lorenzo.dellatti@ausl.re.it (L.D.); federica.mereu@ausl.re.it (F.M.); massimiliano.fabozzi@ausl.re.it (M.F.); 2 Pathology Unit, Reggio Emilia Local Agency—IRCCS Advanced Technologies and Care Models in Oncology, 42123 Reggio Emilia, Italy; magda.zanelli@ausl.re.it (M.Z.); andrea.palicelli@ausl.re.it (A.P.); 3 Department of General Surgery, Azienda USL of Piacenza, 29121 Piacenza, Italy; m.giuffrida@ausl.pc.it; 4 Department of Oncology-Hematology, Azienda USL of Piacenza, 29121 Piacenza, Italy; e.orlandi@ausl.pc.it

**Keywords:** pancreatic stump, mesh wrapping, fistula, pancreatectomy, pancreatic cancer, outcomes

## Abstract

*Background and Objectives*: Post-Operative Pancreatic Fistula (POPF) is reported among 13% to 64% of cases following a distal/left pancreatectomy (D/LP). Many efforts aim to prevent the onset of POPF or reduce its clinical impact. This meta-analysis sought to provide data by assessing POPF rates among patients undergoing D/LP for benign or malignant pancreatic diseases, with or without pancreatic stump mesh wrapping. *Materials and Methods*: We undertook a systematic review following the PRISMA guidelines, alongside the Cochrane Handbook for Systematic Reviews of Interventions. We evaluated the certainty in the evidence using the GRADE approach for the following key outcomes: overall POPF and clinically relevant POPF. PubMed/MEDLINE, Web of Science, and Scopus were employed to retrieve relevant papers. Pooled analysis was carried out employing RevMan Version 5.4.1. *Results*: Among the 8 comparative studies considered (1042 subjects: 430 Wrapping Mesh Group (WMG) versus 612 control group (CG)), seven were retrospective observational studies and one was a randomized controlled trial. Polyglycolic acid (PGA) mesh was used in 7 studies, except for one, who used a polyglactin mesh. Regarding the primary outcomes, meta-analysis showed lower rates of Overall POPF (Ov-POPF) (OR: 0.57, 95% CI: 0.37, 0.88; *p* = 0.01) and clinically relevant POPF (CR-POPF) (OR: 0.33, 95% CI: 0.21, 0.50; *p* < 0.00001) in the WMG. Moreover, the WMG also showed a decrease in Estimated Blood Loss (EBL) (MD: −43.11, 95%, CI: −63.20, −23.02, *p* < 0.0001), a shorter period with surgical drain (MD: −9.66, 95% CI: −17.99, −1.34, *p* = 0.02) and a decreased length of hospital stay (MD: −4.60, 95%, CI: −7.83, −1.36, *p* = 0.005). *Conclusions*: Our meta-analysis showed that wrapping the pancreatic stump with mesh is associated with lower rates of overall POPF and clinically relevant POPF, lower EBL, a shorter period with the surgical drain and reduced hospital stay duration. There is a need for high-quality methodological research to identify the risk factors for the onset of POPF and to evaluate and compare the results of various surgical approaches used to reduce its rate and associated morbidity.

## 1. Introduction

In 2025, the American Cancer Society estimates around 67,440 incident cases of pancreatic cancer per year in the United States, leading to approximately 51,980 deaths per year [1]. Surgery is the cornerstone of treatment for benign and malignant pancreatic diseases. The future outlook, particularly for pancreatic adenocarcinoma, is expected to show an increase in incidence [2]. Distal/left pancreatectomy (D/LP) is the standard surgical procedure for lesions in the body and tail of the pancreas, whether benign or malignant.

Post-Operative Pancreatic Fistula (POPF), following a distal/left pancreatectomy, occurs in 13% to 64% of cases [3,4,5,6]. During 2005, the International Study Group of Pancreatic Fistula (ISGPF) established the first definition of POPF [7]. POPF were further classified according to clinical criteria into three grades, A, B, and C, based on the severity of specific adverse events. These categories were subsequently revised in 2016 [6]. In the revised criteria, grade A as it was previously defined is regarded as a biochemical leak (BL), while POPF includes grades B and C (clinically relevant POPF (CR-POPF)). A CR-POPF is considered a drainage volume of any measurable amount of fluid with a serum amylase concentration three times higher than the upper normal serum amylase concentration, directly associated with a clinically relevant complication or manifestation. This definition is now widely accepted and used. POPF stands as one of the most frequent and significant adverse events, posing a challenge in post-operative management. This leads to an increased risk of wound infections, intra-abdominal abscesses, hemorrhage, sepsis and/or death, affecting both the patient and the entire healthcare system, causing delays in access to adjuvant therapy, prolonged hospital stays and increased healthcare costs. Therefore, many of the efforts undertaken by the scientific/surgical community are aimed at preventing the onset of POPF or reducing its clinical impact. To prevent POPF, various methods are employed [8]. However, to our knowledge, the literature has yet to identify a more advantageous method than the others for reducing POPF [8]. In recent years, the use of polyglycolic acid (PGA) mesh has been introduced, but its impact remains unclear. Currently, it remains necessary to evaluate whether the benefits of wrapping the pancreatic stump with a mesh, after D/LP (both with minimally invasive and open techniques), translate into a reduction in the occurrence of POPF and CR-POPF.

A systematic review of the literature and a meta-analysis of studies were conducted, with the hypothesis that wrapping the pancreatic stump with a mesh would reduce the number of postoperative pancreatic fistulas (both overall and clinically relevant, as primary outcomes) compared to control groups receiving standard treatment.

## 2. Materials and Methods

Throughout our meta-analysis, we complied with both the Preferred Reporting Items for Systematic Reviews and Meta-Analyses (PRISMA) statement and guidelines [9], alongside the Cochrane Handbook for Systematic Reviews of Interventions [10]. It was not required to gain authorization from the Ethics Committee and written consent from study participants, as our meta-analysis was conducted using previously released studies, without any modifications to the original records on the individuals involved.

### 2.1. Search Strategy

In April 2025, we employed the PubMed/MEDLINE, Web of Science (Science and Social Science Citation Index), and Scopus databases to retrieve manuscripts suitable for our meta-analysis. The details of the non-MeSH/MeSH terms used are provided in the Supplementary Materials (see Supplementary Materials, File S1. Search Strategy).

Additionally, the reference lists of the main studies were reviewed individually, and the papers retrieved through Pubmed’s ‘related articles’ function were evaluated. By allowing any language for publication, full-text manuscripts in other linguistic forms besides English, with titles/abstracts that fulfilled the inclusion criteria, were electronically translated and then assessed.

### 2.2. Inclusion and Exclusion Criteria

In this meta-analysis, all types of studies with group comparisons (case series, case–control studies, cohort studies, controlled clinical trials, and randomized clinical trials) involved patients who either underwent or did not undergo (control group) wrapping of the remnant pancreas with mesh after distal/left pancreatectomy for benign or malignant lesions of the left pancreas. We included all mesh types and conducted subgroup analyses for each mesh type. We did not include the following types of articles/publications: abstracts, posters, letters to the editor, study protocols, non-human studies, editorials, clinical cases, and systematic reviews and/or meta-analyses published before. Nevertheless, we have examined previously released systematic reviews or meta-analyses to locate any comparative studies that may have been omitted in our systematic research [11].

### 2.3. Outcomes

The main outcomes were the overall occurrence of postoperative POPF and CR-POPF after D/LP. POPF was classified according to the guidelines established by the International Study Group on Pancreatic Fistula (ISGPF) [6,7]. CR-POPF was classified as a grade B or C POPF. As secondary outcomes, we evaluated the short-term intra-operative and postoperative outcomes. Within the intra-operative outcomes, we recognized and examined the measures listed below: overall operative time (overall-OP) and estimated blood loss (EBL). Regarding post-operative outcomes, we have recognized and assessed: POPF grade A/BL, POPF grade B, POPF grade C, overall major postoperative complications rate (Clavien-Dindo or CD ≥ III), time required to remove the drain, length of hospital stay and readmission.

### 2.4. Data Extraction

Two autonomous evaluators, AM and MZ, conducted all stages of data identification, selection, quality assessment, and extraction. The discrepancies detected throughout the data evaluation and selection process were resolved through a collaborative discussion between the two reviewers, ultimately leading to a consensus. To enhance data precision, a double-blind method was implemented, resulting in an elevated and reliable level of agreement among assessors (Kappa = 0.91). An impartial assessor (FM) has comprehensively assessed all the collected data. By employing the Rayyan web app for systematic reviews (https://www.rayyan.ai/, accessed on 20 April 2025), the selected studies were initially screened according to their title and abstract, and then the full texts were examined [12]. The Rayyan web tool was also employed to screen for and then discard duplicate articles manually. When two or more research papers had patient cohorts that partially coincided during the study period, the most up-to-date report was selected for the pooled analysis.

### 2.5. Quality Assessment

The Cochrane Risk-of-Bias tool for randomized trials (Version 2) (RoB 2) and the Risk Of Bias In Non-randomized Studies—of Interventions (Version 2) (ROBINS-I, V2) were adopted by the two impartial evaluators to appraise the quality of the selected research [13,14].

### 2.6. Statistical Analysis

To perform our meta-analysis, we utilized the Review Manager (RevMan) [Computer program] version 5.4.1, provided by the Cochrane Collaboration in 2020. Whenever dichotomous results were observed, we adopted the corresponding odds ratios (OR) and 95% confidence intervals (CIs), following the Mantel-Haenszel (MH) method. For continuous outcomes, we adopted weighted mean differences (WMD) and the corresponding 95% CIs, which follow the inverse-variance method. Where no mean or standard deviation (SD) for an endpoint was provided, we relied on Wan and Luo’s formulas, with the reported median range and/or interquartile range (IQR) as the starting point to calculate the required data [15,16]. Additionally, if a study had separate sample sizes, means, and SDs for two or more subgroups within each intervention group, the Cochrane formula was employed to derive a single sample size, mean, and SD for each intervention group [10]. To determine statistical heterogeneity, we used I^2^ statistics, which assign heterogeneity values to the following categories: low (<25%), medium (25–50%), and high (>50%). Considering the disparities in the general features of the sample, as well as in the surgical strategies, a random-effects model was employed by default in all statistical analyses with a statistical significance of *p* < 0.05. Sensitivity evaluation was carried out using the leave-one-out method to assess the impact of each study on the overall effect-size estimate and significant studies. Additionally, subgroup analyses were conducted to determine the impact on the outcomes of interest for all mesh used to wrap the pancreatic stump and for all specific surgical methods used in the control group. The assessment of publication bias was conducted using the Egger test, exclusively in meta-analyses that included 10 or more studies.

### 2.7. Summary of Findings and Assessment of the Certainty of the Evidence

#### 2.7.1. Summary of Findings Tables

We prepared summary of findings (SoF) tables to set the group that wraps the pancreatic stump with mesh after distal/left pancreatectomy against the standard or alternative treatment [17].

We reported the listed outcomes in the SoF tables:

- Overall Post-Operative Pancreatic Fistula
- Clinically Relevant Post-Operative Pancreatic Fistula

#### 2.7.2. Assessment of the Certainty of the Evidence

We applied the GRADE approach to evaluate the certainty of the evidence for the outcomes mentioned in the foregoing chapter. The GRADE approach uses five domains (risk of bias, consistency of effect, imprecision, indirectness, and publication bias) to grade the certainty of the body of evidence for each outcome. Two authors (AM and FM) separately evaluated the certainty of each outcome. Disputes were handled by a third author (MZa).

If serious or very serious issues were identified in any of the areas mentioned above, our certainty in the evidence was reduced by one, two, or three levels, as follows:

- Study limitations: moderate (−1) or serious (−2) risk of bias; in addition, for non-randomized studies (−3) for critical risk of bias
- Inconsistency: serious (−1) or very serious (−2) inconsistency
- Indirectness: serious (−1) or very serious (−2) uncertainty about directness
- Imprecision: serious (−1) or very serious (−2) imprecise or sparse data
- Publication bias: serious (−1) or very serious (−2) probability of reporting bias

The criteria for assigning evidence levels, as used by the GRADE system, are detailed in the Supplementary Materials (see Supplementary Materials, File S2. Summary of Findings 1).

For these assessments, we followed the current GRADE guidelines [17].

## 3. Results

### 3.1. Search Results

**Figure 1:**
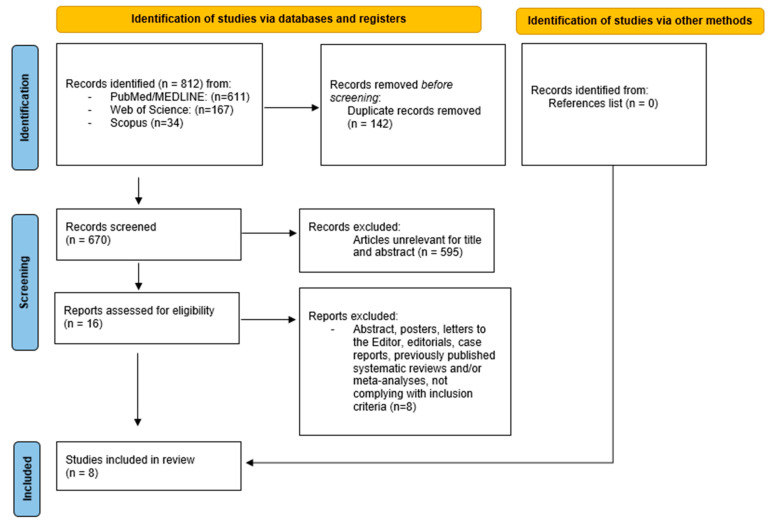
PRISMA flow chart of the literature search.

After carrying out our comprehensive literature review, we identified 812 potentially relevant studies (PubMed/MEDLINE: 611 records; Web of Science: 167 records; Scopus: 34 records) Figure 1. Following a review of the publications to screen for duplicates, we excluded 142 articles. Furthermore, 595 papers were excluded after an in-depth evaluation of their titles and abstracts. Accordingly, 16 texts were identified for a rigorous review. As 8 articles did not satisfy the eligibility criteria, only 8 comparative articles were considered for qualitative and quantitative synthesis, as they fulfilled the necessary criteria [18,19,20,21,22,23,24,25]. Once the other sources (reference list) were analyzed, no further records were retrieved.

### 3.2. Quality of Studies

**Figure 2:**
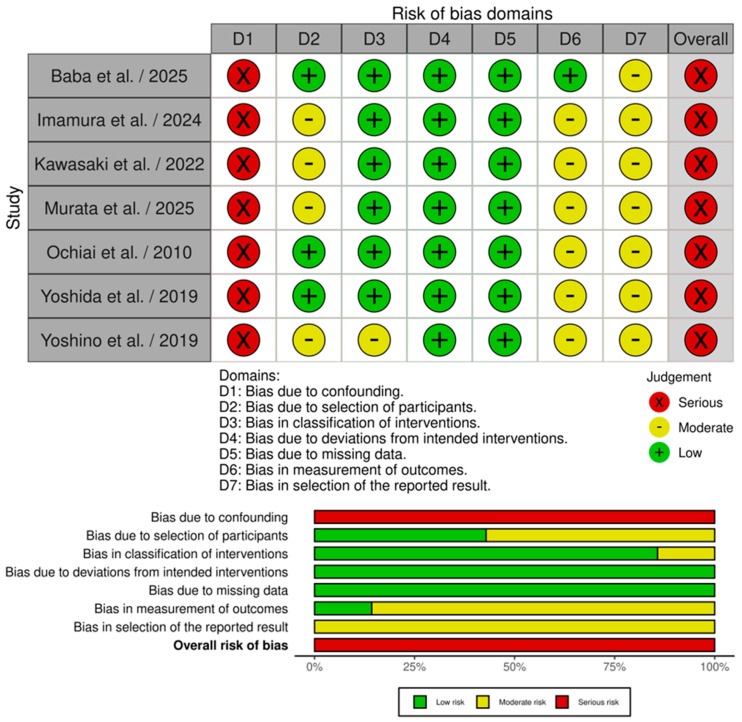
ROBINS-I Traffic-light plot and Summary Plot [18,19,21,22,23,24,25].

According to ROBINS-I V2, seven non-randomized studies revealed “serious” overall bias [18,19,21,22,23,24,25] (Figure 2).

**Figure 3:**
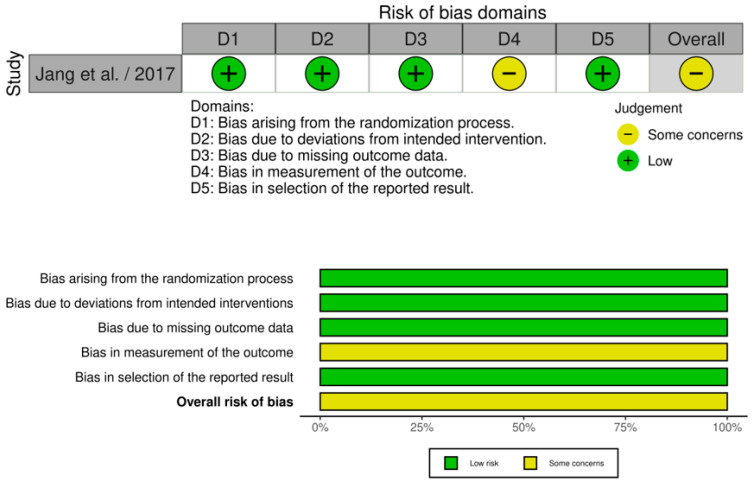
RoB2 Traffic-light plot and Summary Plot [20].

According to RoB 2, one study revealed “some concerns” bias [20] (Figure 3).

### 3.3. Study and Population Characteristics

Table 1 provides details of the included studies and cohorts. Among the eight studies included in the systematic review, seven were retrospective observational studies [18,19,21,22,23,24,25] and one was a randomized controlled trial [20]. They came from Japan [18,19,21,22,23,24,25] and South Korea [20]. The recorded observation period lasted approximately 21 years (2003–2024), Table 1. The overall cohort totaled 1042 patients, with study group sizes ranging from 17 to 123. Five studies reported data on the sex of patients: 48.9% of patients were male (345/706) [18,19,20,22,24]. Data from 5 studies showed that the mean age of the cohorts in each study assessed was between 54.5 and 68.5 years [18,19,20,22,24]. Data from 4 studies showed that mean BMI of the cohorts in each study evaluated fell between 21.3 and 23.1 kg/m^2^ [18,19,22,24], Table 1. The Wrapping Mesh Group (WMG) population comprised 430 patients (41.3%) with study cohort sizes spanning 17 to 115 [18,19,20,21,22,23,24,25]. 52.4% patients were male (143/273) [18,19,20,22,24]. The mean age of the subjects in each study reviewed fell within the range of 59.9 to 68.5 years old [18,19,20,22,24]. The mean BMI of the subjects in each study examined spanned from 21.3 to 23.1 kg/m^2^ [18,19,22,24]. PGA mesh was used in 7 studies [18,19,20,21,22,23,25], except for Yoshida et al., who used a polyglactin mesh (Vicryl mesh; Ethicon, Inc., Somerville, NJ, USA) [24], Table 1. Table 1 also lists the specific surgical methods used, in addition to the application of the mesh, by the various experimental groups, for the handling of the pancreatic remnant with the purpose of reducing POPF. Specifically: six studies reported the use of a sealant in combination with mesh [18,19,20,21,23,25], while two reported the use of a transpancreatic mattress suture [22,24]. Three studies have reported the use of a reinforced suture for pancreatic transection [18,21,23], Table 1. The population of control groups (CG) consisted of 612 patients (58.7%) with study sample sizes ranging from 32 to 123 [18,19,20,21,22,23,24,25]. 46.7% patients were male (202/433) [18,19,20,22,24]. The mean age of the cohorts in each study assessed ranged from 54.5 to 67.6 [18,19,20,22,24]. The mean BMI of the subjects in each study assessed spanned 21.7 to 22.4 kg/m^2^ [18,19,22,24]. Supplementary Materials, Table S1 lists: the type of surgical approach used for D/LP (open, laparoscopic or robotic), whether or not the D/LP was with preservation of the spleen, whether intra-abdominal surgical drainage was placed, the histology of the lesion.

Table 2 and Table 3 show the intra-operative (overall-operative time, estimated blood loss) and post-operative (overall-POPF, CR-POPF, POPF grade A/BL, POPF grade B, POPF grade C, time to remove the drain, overall major postoperative complications rate (Clavien-Dindo or CD ≥ III), length of hospital stay and readmission) outcomes. overall-OP was reported by 5 studies [18,19,20,22,24], EBL was reported by 5 studies [18,19,20,22,24]; overall-POPF was reported by all the included studies [18,19,20,21,22,23,24,25]; CR-POPF was reported by 7 studies [18,19,20,21,22,23,24]; POPF grade A/BL was reported by 7 studies [18,19,20,21,22,23,24]; POPF grade B was reported by 4 studies [19,20,22,24]; POPF grade C was reported by 4 studies [19,20,22,24]; time to remove the drain was reported by 2 studies [18,21]; overall major postoperative complications rate (Clavien-Dindo or CD ≥ III) was reported by 4 studies [18,19,22,24]; length of hospital stay was reported by 6 studies [18,19,20,21,22,24] and readmission was reported by 4 studies [18,19,22,24].

### 3.4. Meta-Analyses Results

#### 3.4.1. Overall Post-Operative Pancreatic Fistula

##### Wrapping Mesh Group vs. Control Group

**Figure 4:**
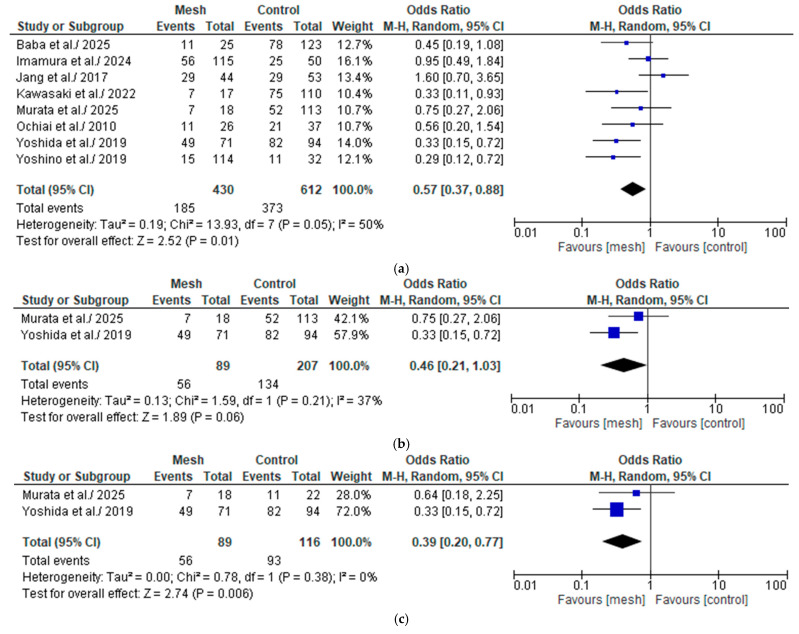

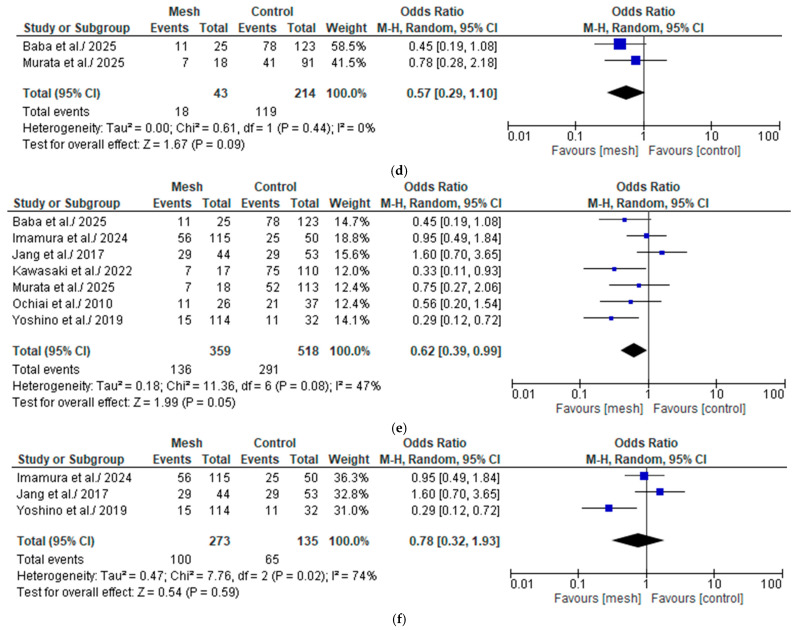
(**a**) Forest plot comparing overall post-operative pancreatic fistula between the Wrapping mesh group and Control group. (**b**) Forest plot comparing overall post-operative pancreatic fistula between the Wrapping mesh group (only with transpancreatic mattress suture) vs. Control group. (**c**) Forest plot comparing overall post-operative pancreatic fistula between the Wrapping mesh group vs. Control group (only with hand-sewn closure technique). (**d**) Forest plot comparing overall post-operative pancreatic fistula between the Wrapping mesh group vs. Control group (only with reinforced stapler suture closure). (**e**) Forest plot comparing overall post-operative pancreatic fistula between the Wrapping mesh group (only with PGA mesh) vs. Control group. (**f**) Forest plot comparing overall post-operative pancreatic fistula between the Wrapping mesh group (only with PGA mesh and without reinforced stapler suture closure) vs. Control group [18,19,20,21,22,23,24,25].

The overall-POPF was examined by all eight studies included in our meta-analysis, involving 1042 patients: 430 in the WMG and 612 in the CG [18,19,20,21,22,23,24,25], as shown in Figure 4a. Following an examination of the pooled results on the overall-POPF, based on the meta-analysis, a statistically significant difference was identified within the groups under review [Odds Ratio (OR): 0.57, 95% confidence interval (CI): 0.37, 0.88; *p* = 0.01]. The reported I^2^ value was 50% (medium heterogeneity), with a *p*-value of 0.05, a result that was statistically significant. We downgraded the certainty of the evidence to one level for serious limitations in study design or execution (risk of bias) and another for serious inconsistency due to substantial statistical heterogeneity with an I^2^ of 50%, with a *p*-value of 0.05 (see Supplementary Materials, File S2. Summary of Findings 1).

##### Wrapping Mesh Group (Only with Transpancreatic Mattress Suture) vs. Control Group

The overall-POPF was assessed by two of the eight studies considered in our meta-analysis, comprising 296 patients: 89 in the WMG (only with transpancreatic mattress suture) and 207 in the CG group [22,24], as shown in Figure 4b. Following an examination of the pooled results on the overall-POPF, based on the meta-analysis, no statistically significant difference was identified within the groups under review [Odds Ratio (OR): 0.46, 95% confidence interval (CI): 0.21, 1.03; *p* = 0.06]. The reported I^2^ value was 37% (medium heterogeneity), with a *p*-value of 0.21, a result that was not statistically significant.

##### Wrapping Mesh Group vs. Control Group (Only with Hand-Sewn Closure Technique)

The overall-POPF was assessed by two of the eight considered in our meta-analysis, comprising 205 patients: 89 in the WMG and 116 in the CG group (only with hand-sewn closure technique) [22,24], as shown in Figure 4c. Following an examination of the pooled results on the overall-POPF, based on the meta-analysis, a statistically significant difference was identified within the groups under review [Odds Ratio (OR): 0.39, 95% confidence interval (CI): 0.20, 0.77; *p* = 0.006]. The reported I^2^ value was 0% (low heterogeneity), with a *p*-value of 0.38, a result that was not statistically significant.

##### Wrapping Mesh Group vs. Control Group (Only with Reinforced Stapler Suture Closure)

The overall-POPF was assessed by two of the eight studies considered in our meta-analysis, comprising 257 patients: 43 in the WMG and 214 in the CG (only with reinforced suture closure) [18,22] Figure 4d. Following an examination of the pooled results on the overall-POPF, based on the meta-analysis, no statistically significant difference was identified within the groups under review [Odds Ratio (OR): 0.57, 95% confidence interval (CI): 0.29, 1.10; *p* = 0.09]. The reported I^2^ value was 0% (low heterogeneity), with a *p*-value of 0.44, a result that was not statistically significant.

##### Wrapping Mesh Group (Only with PGA Mesh) vs. Control Group

The overall-POPF was assessed by seven of the eight studies considered in our meta-analysis, comprising 877 patients: 359 in the WMG (only with PGA mesh) and 518 in the CG [18,19,20,21,22,23,25], as shown in Figure 4e. Following an examination of the pooled results on the overall-POPF, based on the meta-analysis, a statistically significant difference was identified within the groups under review [Odds Ratio (OR): 0.62, 95% confidence interval (CI): 0.39, 0.99; *p* = 0.05]. The reported I^2^ value was 47% (medium heterogeneity), with a *p*-value of 0.08, a result that was not statistically significant.

##### Wrapping Mesh Group (Only with PGA Mesh and Without Reinforced Stapler Suture Closure) vs. Control Group

The overall-POPF was assessed by three of the eight studies considered in our meta-analysis, comprising 408 patients: 273 in the WMG (only with PGA mesh and without reinforced suture closure) and 135 in the CG [19,20,25], as shown in Figure 4f. Following an examination of the pooled results on the overall-POPF, based on the meta-analysis, no statistically significant difference was identified within the groups under review [Odds Ratio (OR): 0.78, 95% confidence interval (CI): 0.32, 1.93; *p* = 0.59]. The reported I^2^ value was 74% (high heterogeneity), with a *p*-value of 0.59, a result that was not statistically significant.

#### 3.4.2. Clinically Relevant Post-Operative Pancreatic Fistula

##### Wrapping Mesh Group vs. Control Group

**Figure 5:**
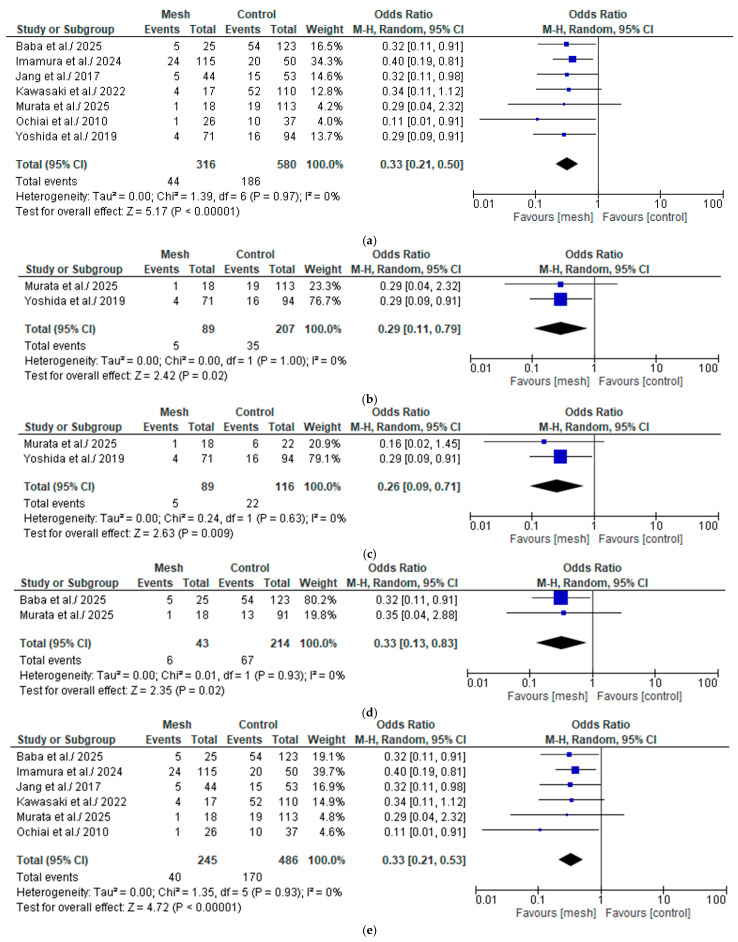

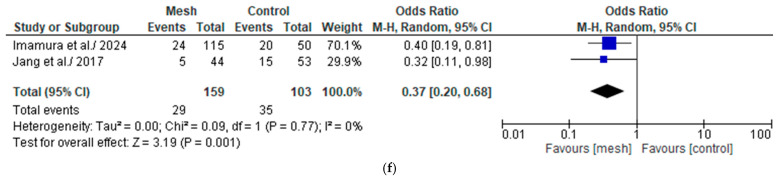
(**a**) Forest plot comparing clinically relevant post-operative pancreatic fistula between the Wrapping mesh group vs. Control group. (**b**) Forest plot comparing clinically relevant post-operative pancreatic fistula between the Wrapping mesh group (only with transpancreatic mattress suture) vs. Control group. (**c**) Forest plot comparing clinically relevant post-operative pancreatic fistula between the Wrapping mesh group vs. Control group (only with hand-sewn closure technique). (**d**) Forest plot comparing clinically relevant post-operative pancreatic fistula between the Wrapping mesh group vs. Control group (only with reinforced stapler suture closure). (**e**) Forest plot comparing clinically relevant post-operative pancreatic fistula between the Wrapping mesh group (only with PGA mesh) vs. Control group. (**f**) Forest plot comparing clinically relevant post-operative pancreatic fistula between the Wrapping mesh group (only with PGA mesh and without reinforced stapler suture closure) vs. Control group [18,19,20,21,22,23,24,25].

The CR-POPF was assessed by seven of the eight studies considered in our meta-analysis, comprising 896 patients: 316 in the WMG and 580 in the CG [18,19,20,21,22,23,24], as shown in Figure 5a. Following an examination of the pooled results on the CR-POPF, based on the meta-analysis, a statistically significant difference was identified within the groups under review [Odds Ratio (OR): 0.33, 95% confidence interval (CI): 0.21, 0.50; *p* < 0.00001]. The reported I^2^ value was 0% (low heterogeneity), with a *p*-value of 0.97, a result that was not statistically significant. We downgraded the certainty of the evidence to one level for serious limitations in study design or execution (risk of bias) (see Supplementary Materials, File S2. Summary of Findings 1).

##### Wrapping Mesh Group (Only with Transpancreatic Mattress Suture) vs. Control Group

The CR-POPF was assessed by two of the eight studies considered in our meta-analysis, comprising 296 patients: 89 in the WMG (only with transpancreatic mattress suture) and 207 in the CG [22,24], as shown in Figure 5b. Following an examination of the pooled results on the CR-POPF, based on the meta-analysis, a statistically significant difference was identified within the groups under review [Odds Ratio (OR): 0.29, 95% confidence interval (CI): 0.11, 0.79; *p* = 0.02]. The reported I^2^ value was 0% (low heterogeneity), with a *p*-value of 1.00, a result that was not statistically significant.

##### Wrapping Mesh Group vs. Control Group (Only with Hand-Sewn Closure Technique)

The CR-POPF was assessed by two of the eight studies considered in our meta-analysis, comprising 205 patients: 89 in the WMG and 116 in the CG (only with hand-sewn closure technique) [22,24] Figure 5c. Following an examination of the pooled results on the CR-POPF, based on the meta-analysis, a statistically significant difference was identified within the groups under review [Odds Ratio (OR): 0.26, 95% confidence interval (CI): 0.09, 0.71; *p* = 0.009]. The reported I^2^ value was 0% (low heterogeneity), with a *p*-value of 0.63, a result that was not statistically significant.

##### Wrapping Mesh Group vs. Control Group (Only with Reinforced Stapler Suture Closure)

The CR-POPF was assessed by two of the eight studies considered in our meta-analysis, comprising 257 patients: 43 in the WMG and 214 in the CG (only with reinforced suture closure) [18,22] Figure 5d. Following an examination of the pooled results on the CR-POPF, based on the meta-analysis, a statistically significant difference was identified within the groups under review [Odds Ratio (OR): 0.33, 95% confidence interval (CI): 0.13, 0.83; *p* = 0.02]. The reported I^2^ value was 0% (low heterogeneity), with a *p*-value of 0.93, a result that was not statistically significant.

##### Wrapping Mesh Group (Only with PGA Mesh) vs. Control Group

The CR-POPF was assessed by six of the eight studies considered in our meta-analysis, comprising 731 patients: 245 in the WMG (only with PGA mesh) and 486 in the CG [18,19,20,21,22,23], as shown in Figure 5e. Following an examination of the pooled results on the CR-POPF, based on the meta-analysis, a statistically significant difference was identified within the groups under review [Odds Ratio (OR): 0.33, 95% confidence interval (CI): 0.21, 0.53; *p* < 0.00001]. The reported I^2^ value was 0% (low heterogeneity), with a *p*-value of 0.93, a result that was not statistically significant.

##### Wrapping Mesh Group (Only with PGA Mesh and Without Reinforced Stapler Suture Closure) vs. Control Group

The CR-POPF was assessed by two of the eight studies considered in our meta-analysis, comprising 262 patients: 159 in the WMG (only with PGA mesh and without reinforced suture closure) and 103 in the CG [19,20], as shown in Figure 5f. Following an examination of the pooled results on the CR-POPF, based on the meta-analysis, a statistically significant difference was identified within the groups under review [Odds Ratio (OR): 0.37, 95% confidence interval (CI): 0.20, 0.68; *p* = 0.001]. The reported I^2^ value was 0% (low heterogeneity), with a *p*-value of 0.77, a result that was not statistically significant.

#### 3.4.3. Overall Operative Time

**Figure 6:**
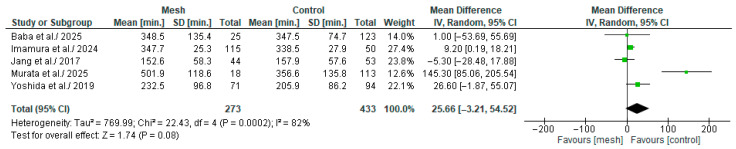
Forest plot comparing overall-operative time between the Wrapping mesh group vs. Control group [18,19,20,22,24].

Overall Operative time (overall-OP) was assessed by five of the eight studies considered in our meta-analysis, comprising 706 patients: 273 in the WMG and 433 in the CG [18,19,20,22,24], as shown in Figure 6. Following an examination of the pooled results on the overall-OP, based on the meta-analysis, no statistically significant difference was identified within the groups under review [MD: 25.66, 95%, CI: −3.21, 54.52, *p* = 0.08]. The recorded I^2^ value was 82% (high heterogeneity), with a *p*-value of 0.0002, a result that was statistically significant.

#### 3.4.4. Estimated Blood Loss

**Figure 7:**
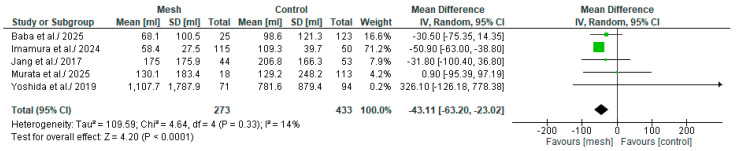
Forest plot comparing Estimated blood loss between the Wrapping mesh group vs. Control group [18,19,20,22,24].

Estimated Blood Loss (EBL) was assessed by five of the eight studies considered in our meta-analysis, comprising 706 patients: 273 in the WMG and 433 in the CG [18,19,20,22,24], as shown in Figure 7. Following an examination of the pooled results on the EBL, based on the meta-analysis, a statistically significant difference was identified within the groups under review [MD: −43.11, 95%, CI: −63.20, −23.02, *p* < 0.0001]. The reported I^2^ value was 14% (low heterogeneity), with a *p*-value of <0.0001, a result that was statistically significant. Using the leave-one-out method, we found that excluding Baba et al., Murata et al. or Yoshida et al. study from the analysis still yielded a statistically significant result. However, excluding Imamura et al. or Jang et al. study, the result persisted in favor of the wrapping mesh group, nonetheless without reaching statistical significance (see Supplementary Materials, Figure S1).

#### 3.4.5. Biochemical Leak/Grade A Post-Operative Pancreatic Fistula

**Figure 8:**
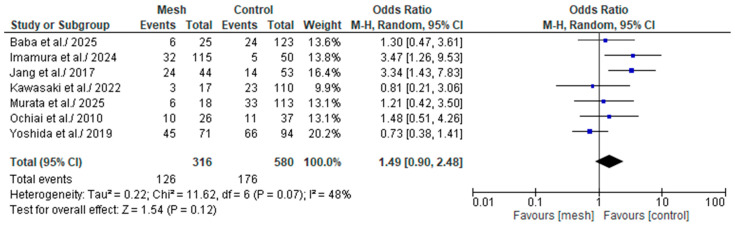
Forest plot comparing Biochemical Leak/Grade A post-operative pancreatic fistula between the Wrapping mesh group vs. Control group [18,19,20,21,22,23,24].

BL/Grade A POPF was assessed by seven of the eight studies considered in our meta-analysis, comprising 896 patients: 316 in the WMG and 580 in the CG [18,19,20,21,22,23,24], as shown in Figure 8. Following an examination of the pooled results on the BL/Grade A POPF, based on the meta-analysis, no statistically significant difference was identified within the groups under review [Odds Ratio (OR): 1.49, 95% confidence interval (CI): 0.90, 2.48; *p* = 0.07]. The recorded I^2^ value was 47% (medium heterogeneity), with a *p*-value of 0.07, a result that was not statistically significant.

#### 3.4.6. Grade B Post-Operative Pancreatic Fistula

**Figure 9:**
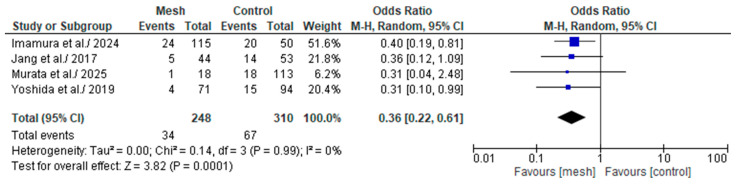
Forest plot comparing Grade B post-operative pancreatic fistula between the Wrapping mesh group vs. Control group [19,20,22,24].

Grade B POPF was assessed by four of the eight studies considered in our meta-analysis, comprising 558 patients: 248 in the WMG and 310 in the CG [19,20,22,24], as shown in Figure 9. Following an examination of the pooled results on the Grade B POPF, based on the meta-analysis, a statistically significant difference was identified within the groups under review [Odds Ratio (OR): 0.36, 95% confidence interval (CI): 0.22, 0.61; *p* = 0.0001]. The recorded I^2^ value was 0% (low heterogeneity), with a *p*-value of 0.99, a result that was not statistically significant.

#### 3.4.7. Grade C Post-Operative Pancreatic Fistula

**Figure 10:**
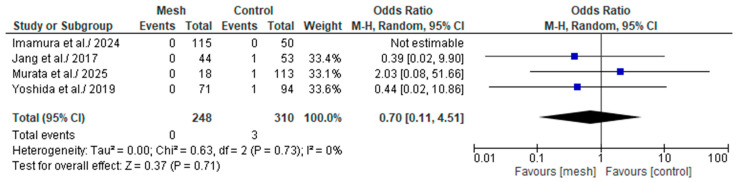
Forest plot comparing Grade C post-operative pancreatic fistula between the Wrapping mesh group vs. Control group [19,20,22,24].

Grade C POPF was assessed by four of the eight studies considered in our meta-analysis, comprising 558 patients: 248 in the WMG and 310 in the CG [19,20,22,24], as shown in Figure 10. Following an examination of the pooled results on the Grade C POPF, based on the meta-analysis, no statistically significant difference was identified within the groups under review [Odds Ratio (OR): 0.70, 95% confidence interval (CI): 0.11, 4.51; *p* = 0.71]. The recorded I^2^ value was 0% (low heterogeneity), with a *p*-value of 0.73, a result that was not statistically significant.

#### 3.4.8. Major (Clavien-Dindo or CD ≥ III) Postoperative Complications

**Figure 11:**
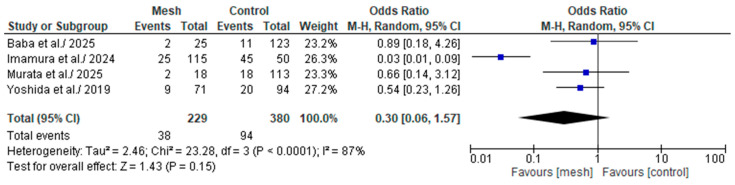
Forest plot comparing Major (Clavien-Dindo or CD ≥ III) postoperative complications between the Wrapping mesh group vs. Control group [18,19,22,24].

Major (Clavien-Dindo or CD ≥ III) postoperative complications was assessed by eight of the eight studies considered in our meta-analysis, comprising 609 patients: 229 in the WMG and 380 in the CG [18,19,22,24], as shown in Figure 11. Following an examination of the pooled results on the CD ≥ III, based on the meta-analysis, a statistically significant difference was identified within the groups under review [Odds Ratio (OR): 0.30, 95% confidence interval (CI): 0.06, 1.57; *p* = 0.15]. The recorded I^2^ value was 87% (high heterogeneity), with a *p*-value of <0.0001, a result that was statistically significant.

#### 3.4.9. Time Required to Remove the Drain

**Figure 12:**
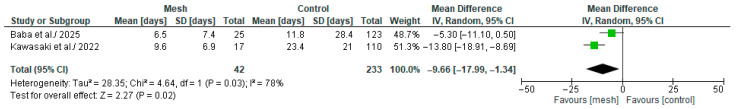
Forest plot comparing Time required to Remove the Drain between the Wrapping mesh group vs. Control group [18,21].

Time required to Remove the Drain (TRD) was assessed by two of the eight studies considered in our meta-analysis, comprising 275 patients: 42 in the WMG and 233 in the CG [18,21], as shown in Figure 12. Following an examination of the pooled results on the TRD, based on the meta-analysis, a statistically significant difference was identified within the groups under review [MD: −9.66, 95% CI: −17.99, −1.34, *p* = 0.02]. The recorded I^2^ value was 78% (high heterogeneity), with a *p*-value of 0.03, a result that was statistically significant.

#### 3.4.10. Length of Hospital Stay

**Figure 13:**
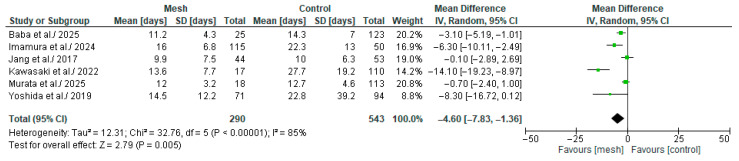
Forest plot comparing Length of Hospital Stay between the Wrapping mesh group vs. Control group [18,19,20,21,22,24].

Length of Hospital Stay (LoHS) was assessed by six of the eight studies considered in our meta-analysis, comprising 833 patients: 290 in the WMG and 543 in the CG [18,19,20,21,22,24], as shown in Figure 13. Following an examination of the pooled results on the LoHS, based on the meta-analysis, a statistically significant difference was identified within the groups under review [MD: −4.60, 95%, CI: −7.83, −1.36, *p* = 0.005]. The recorded I^2^ value was 85% (high heterogeneity), with a *p*-value of <0.00001, a result that was statistically significant.

#### 3.4.11. Readmission

**Figure 14:**
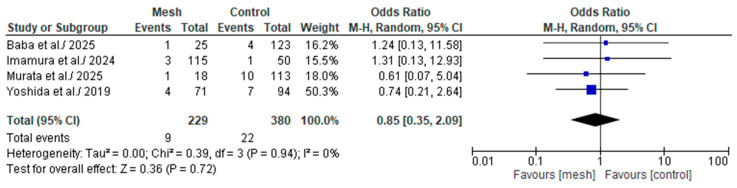
Forest plot comparing readmission between the Wrapping mesh group vs. Control group [18,19,22,24].

Readmission was assessed by four of the eight studies considered in our meta-analysis, comprising 609 patients: 229 in the WMG and 380 in the CG group [18,19,22,24], as shown in Figure 14. Following an examination of the pooled results on readmission, based on the meta-analysis, no statistically significant difference was identified within the groups under review [Odds Ratio (OR): 0.85, 95% confidence interval (CI): 0.35, 2.09; *p* = 0.72]. The recorded I^2^ value was 0% (low heterogeneity), with a *p*-value of 0.72, a result that was not statistically significant.

#### 3.4.12. Publication Bias

As stated in the Cochrane Handbook for Systematic Reviews of Interventions (Version 5.1.0), evaluations for funnel plot asymmetry should be carried out only in meta-analyses comprising 10 or more studies [26]. Due to our meta-analysis comprising eight studies, we did not perform an analysis of publication bias. In fact, fewer studies impair the power of tests to identify the case from real asymmetry [26].

## 4. Discussion

### 4.1. Discussion

The distal/left pancreatectomy, the management of the resulting pancreatic stump, and the treatment of overall postoperative pancreatic complications (which occurs in 10% to 46% of patients [27]) represent a highly complex technical challenge for surgeons in pancreatic surgery, whether for benign or malignant conditions. Pancreatic fistula, among postoperative complications, is one of the most common, remaining a critical weakness of distal/left pancreatectomy. It leads to a poor prognosis resulting from the onset of intra-abdominal abscesses, intra-abdominal bleeding, wound infection, and sepsis. The onset of POPF cannot be completely prevented. The pathogenesis of POPF is multifactorial determined by several factors, both non-modifiable, including age, BMI, neuroendocrine or nonmalignant pathology, pancreatic consistency and thickness, as well as modifiable factors like hypoalbuminenia, absence of epidural anesthesia, concomitant splenectomy, vascular resection, intraoperative fluid balance and blood loss [28]. Even without these risk factors, surgical management plays a significant role in the development of POPF. Despite numerous attempts to implement surgical techniques, new technologies, or materials to minimize the risk of fistula formation, the risk of its occurrence after distal/left pancreatectomy remains high (19,4% of the patients undergoing laparoscopic D/LP developed POPF grade B/C [29,30]). Among the various methods used to prevent POPF, the use of a mesh that wraps around the residual pancreatic stump after a distal/left pancreatectomy has been proposed. This method has been introduced over the past 20 years and has shown effectiveness [31,32,33]. Among the introduced meshes was the polyglycolic acid (PGA) mesh (Neoveil; Gunze), a bioabsorbable recombinant membrane made from a synthetic polymer with a structure similar to that of cellulose. Immediately after insertion, the PGA mesh triggers an inflammatory process and is infiltrated by granulation tissue within 3 weeks. It is then absorbed after 2–3 months [34]. As previously described by Ceonzo et al., in vitro studies on mice have shown that during the degradation of polyglycolic acid (polyglycolic acid mesh), the hemolytic activity of the serum decreases and inflammation increases. This correlates with clinical findings of increased inflammation [35]. Also Polyglactin 910 woven mesh (VICRYL Mesh, Ethicon, Inc.) is widely used in various surgical specialties, including colorectal, cardiothoracic, gynecological, breast, and neurosurgery [36,37,38]. Polyglactin 910 woven mesh is composed of copolymers made from 90% glycolide and 10% L-lactide. It is a synthetic and absorbable mesh with high tensile strength and great flexibility, providing excellent tissue support [39].

This systematic review and meta-analysis was conducted to determine whether the wrapping of the mesh around the pancreatic stump is responsible for a lower rate of postoperative pancreatic fistula.

In our meta-analysis, we included a total of eight studies, one of which was an RCT, while the others were retrospective observational studies. In total, 1042 patients were analyzed: 430 in the wrapping mesh group and 612 in the control group. In wrapping mesh group, the most widely considered mesh was the PGA, which was present in seven out of eight studies, while only one study analyzed the effect of the polyglactin 910 mesh. Analyzing our results regarding overall-POPF, the fistula rate in the wrapping mesh group is 42% (185/430), while in the control group it is 60.9% (373/612). The data was consistent with the information reported in the literature and previously mentioned [3,4,5,6]. When evaluating overall POPF, we observe that the wrapping mesh group has a lower rate of pancreatic fistulas compared to the control group. The observed data is statistically significant, with moderate heterogeneity.

To mitigate the presence of potential confounding factors, we compared the studies by grouping them into subcategories. The statistically significant result showing a lower overall POPF rate in favor of the wrapping mesh group was also confirmed in studies using only PGA mesh in the wrapping mesh group, as well as in those with the control group using only the hand-sewn closure technique. In the wrapping mesh group subcategory “only with transpancreatic mattress suture” and in the comparison between wrapping mesh group (“all mesh”) and the control group subcategory “with only reinforced stapler suture closure”, a trend favoring the wrapping mesh group was observed, although it was not statistically significant. Only in the comparison between the wrapping mesh group subcategory “only PGA mesh + sealant without reinforced stapler suture closure” and the control group, no supportive evidence for wrapping mesh group was observed, showing no statistically significant differences between the two groups. For practical and daily application, even when considering subcategory analyses, it is evident that the protective effect of the mesh is maintained, including with the PGA mesh. Statistical significance is not achieved in analyses that examine a limited number of studies and patients. In particular, when comparing the wrapping mesh group subcategory (only PGA mesh + sealant without reinforced stapler suture closure) to the control group, the included studies also include Imamura et al. and Jang et al. both of these studies present results favoring the control group, even in the “BL/Grade A POPF” subcategory, which also affects overall POPF. In the analysis of the CR-POPF (which does not include the research by Yoshino et al. due to the lack of individual patient data), we observe a consistent and statistically significant result in support of the wrapping mesh group, both in the overall comparison and in the comparison between subcategories. These data suggest that using mesh to wrap the pancreatic stump has a protective effect, reducing both the number of POPFs (overall POPF) and the significant clinical symptoms of these complications, thereby decreasing the number of CR-POPFs. Furthermore, the reduction in CR-POPF appears to be effective even in subgroup analyses, where we have minimized the interference of confounding factors. The analyses of the CR-POPF rate also show a low and not statistically significant level of heterogeneity.

For completeness in our analysis, we also compared the wrapping mesh group and control group for each grade of POPF, using the ISGPF classification. In the comparison between BL and Grade A POPF, although a statistically significant difference was not achieved, there is an observable trend leaning towards the control group. In contrast, in grade B POPF, a statistically significant difference is achieved in favor of the wrapping mesh group. In the case of grade C POPF, although no grade C POPF events were observed in the wrapping mesh group, the limited number of events recorded in both groups prevented a statistically significant difference from being achieved. The analyzed data are limited, as not all studies provided their data disaggregated into the three degrees of POPF. Instead, they only provided BL/grade A POPF and CR-POPF. Therefore, for the interpretation of these data, please refer to the previous paragraphs of this discussion. However, it is important to note that all studies have cataloged the POPF according to the ISGPF guidelines. For studies with an extended data collection period, the reliability of the data collection may have been affected by the use of either the first or second version of the ISGPF guidelines. As previously mentioned, this is a limitation of our meta-analysis. When comparing techniques aimed at reducing the occurrence of an event, agreement in defining and classifying the data is a fundamental prerequisite and has been a major bias in many studies.

Regarding the other secondary outcomes, our meta-analysis showed that using a mesh to wrap the pancreatic stump appears to have a lower estimated blood loss, reduce the time the drain remains in place, and shorten the length of hospital stay. When these results are translated into clinical practice, a possible interpretation is that reducing the rate of POPF (especially CR-POPF) will accelerate the removal of drainage and the patient’s discharge home. The presence of a clinically significant POPF can lead to a prolonged need for drainage, which in turn results in a longer hospital stay. These results would be achieved without extending the overall operative time. Actually, we observed a trend towards shorter operative times in the control group, but this finding was not statistically significant. However, these data must be interpreted with caution and cannot be generalized. Due to the lack of individual patient data, we were unable to verify whether the duration of surgery was influenced not only by the application of the mesh but also by other confounding factors, such as: the technique used to manage the pancreatic stump, the type of surgical approach (open, laparoscopic, or robotic), the surgical team’s experience and track record, the indication for performing the DP/L (malignant or benign pathology), and whether an associated splenectomy was performed. These influencing factors not only affect the overall operating time but also impact the estimated blood loss. Furthermore, in the analysis of the Estimated blood loss, which was found to be lower in the wrapping mesh group (a statistically significant result), it is important to consider the significant impact of two single study [19,20] in favor of wrapping mesh group, which ultimately determines the overall result of the aggregate analysis. In fact, as previously analyzed, excluding one of these two studies results in the lack of significance of the result (see Supplementary Materials). Regarding the removal of the drainage, although it was faster in the wrapping mesh group (a statistically significant result), the analysis is not easily generalizable due to the presence of only two studies and the potential variability in drainage management among operators and during the data collection period within each study. All studies reported the placement of the drain during the surgical procedure, but the criteria for removal were not always specified. Even when they were, the surgical team’s adherence to these criteria was not detailed. This significant interference from confounding factors can be attributed to the type of studies examined (all retrospective except for one RCT) and to the fact that the outcome of the studies involved the rate of POPF, not the management of drainage after DP/L. In terms of Major (Clavien-Dindo or CD ≥ III) postoperative complications and readmissions, although a trend favoring wrapping mesh group was observed, no statistically significant difference was found. In this case as well, the interpretation of these data must be approached with caution and cannot be generalized.

Despite the limitations in the studies included in our meta-analysis, our results confirm the findings previously presented in the literature, expanding the number of studies and patients included [11,40]. The application of mesh to wrap the pancreatic stump is a relatively new management technique. The mesh wrapped around the pancreatic stump triggers an inflammatory response due to its high resistance, absorbability, and sealing effect on the pancreatic stump. This plays a crucial role in lowering the incidence of pancreatic fistula.

In a 2020 systematic review and meta-analysis on the effect of PGA mesh in preventing pancreatic fistula (both for pancreatoduodenectomy (PD) and DP), Zhang et al. demonstrated a protective effect of PGA mesh application compared to the control group [11]. The result in favor of the PGA group was confirmed in both the subgroup analyses for PD and DP. In the analysis of the group undergoing DP, similar to our results, a statistically significant protective effect was observed in the placement of PGA mesh in CR-POPF. However, this effect was not detected in BL/Grade A POPF, where a protective effect was instead found in the control group. Zhang et al. included both patients who used PGA as reinforcement in stapler closure and those who had the mesh wrapped around the pancreatic stump. We believe that including these two categories of pancreatic stump management (reinforced stapler suture closure and PGA mesh wrapping) was not comparable, as they have different effects and inflammatory responses on the pancreatic stump. Furthermore, their effects could be cumulative [18,21]. Therefore, in our meta-analysis, we included only studies that primarily analyzed the protective effect of the mesh wrapping on the pancreatic stump. We also made efforts to limit the interference of confounding effects by evaluating subcategories (where individual patient data were available). The results, despite the previously mentioned limitations, confirm a reduction in the rate of POPF (especially CR-POPF) in wrapping mesh group. Zhang et al., in their meta-analysis, included only PGA mesh, without considering other types of mesh used in clinical practice, such as polyglactin. In our study, we included all types of mesh and set out to perform subgroup analyses by mesh type, where individual patient data were available. Specifically, out of the eight included studies, seven used a PGA mesh and only one used a polyglactin mesh. The statistically significant results in favor of wrapping mesh group were also confirmed in the “PGA mesh only” subcategory, indicating no distorting effect from the single study using polyglactin [24].

In a 2025 umbrella review, Teng et al. found that the PGA mesh was effective in reducing the incidence of pancreatic fistulas [40]. Furthermore, they observed, with moderate-quality evidence, that the fibrin sealant patch did not influence the rate of POPF. They also reported, with evidence ranging from low to very low quality, that ultrasonic dissection and prophylactic transpapillary pancreatic stenting can decrease the rate of overall POPF after DP/L. Minimally invasive-spleen-preserving distal pancreatectomy (MI-SPDP), reinforced stapler, no drainage, and early removal of the drainage tube may decrease the rate of CR-POPF after D/LP. These data are partially consistent with the 2020 consensus on the management of the pancreatic transection plane after DP/L, published by Miao et al. on behalf of the International Study Group of Pancreatic Surgery (ISGPS) [8].

The ISGPS has established strong consensus on the recommendations below: there was no difference in the POPF rate after D/LP between the hand-sewn and stapler techniques; a stapling technique was not suitable for all cases of left pancreatectomy; the use of an energy-based tissue sealant, a chemical sealant device, or a combination of these did not affect the POPF rate; there was no difference in the postoperative pancreatic fistula rate between open, laparoscopic, or robotic approaches; and there are one or more clinically important, patient-related risk factors associated with the POPF rate. There was weak or conditional agreement on the use of prophylactic somatostatin analogs, stents, stump closure, stump anastomosis, and the role of abdominal drains. As previously pointed out by Teng et al., in the ISGPS consensus, the PGA mesh wrapping the pancreatic stump after DP/L was not considered a stand-alone technique. This was likely due to the technique being relatively innovative and the limited data available upon the article’s publication [40].

Although the results of our meta-analysis support the use of mesh to wrap the pancreatic stump after DP/L, the generalization of the data and the subsequent recommendation of mesh placement as a surgical practice still require further strong and robust scientific evidence, based on randomized and large-scale studies. Through large-scale studies, it would be desirable to assess the pro-inflammatory effect of the mesh on the pancreatic stump, which could potentially hinder and complicate surgery in the event of re-operation due to complications. Furthermore, we believe that the risk of loco-regional dissemination in the event of malignant recurrence at the pancreatic margin should also be analyzed and given serious consideration. In the included studies, no adverse or undesirable events were reported during the placement process or in the postoperative period following mesh placement.

Although our analyses considered many confounding factors, some could not be isolated due to the lack of individual patient data. For example, it was not possible to perform subcategory analyses based on the indication (whether benign or malignant) and the surgical approach. Therefore, we could not determine if the reduction in the POPF rate due to the mesh was partly attributable to the use of the minimally invasive technique, as reported by Teng et al. [40], which, however, was inconsistent with the ISGPS consensus [8]. As reported by Ecker et al. in a retrospective analysis of 2026 distal pancreatectomy, the risk factors associated with CR-POPF were: age < 60 years, obesity, hypoalbuminemia, absence of epidural anesthesia, neuroendocrine or nonmalignant pathology, concomitant splenectomy, and vascular resection [28]. Unfortunately, even in this case, the impact of these confounding factors in our analyses could not be measured due to the lack of individual patient data. Other factors that, due to the lack of individual data, could not be analyzed in our meta-analysis were: the type of cartridge used for the closure of the pancreatic stump with a mechanical stapler, the thickness and consistency of the pancreas, the routine use of drainage, and the use of somatostatin analogues during the intra- and/or postoperative phase. Regarding the thickness of the pancreas, the literature indicated that it influenced the choice of both the transection method and the closure technique. The ISGPS consensus strongly agreed on not employing a stapling technique in any case of D/LP. In fact, the literature indicates that the thickness and firmness of the pancreas may play a role in the success of stapler transection during D/LP [8,41,42,43]. A thicker pancreas seems to correlate with a higher risk of overall POPF or CR-POPF after transection with the stapler, but not with different surgical strategies. Currently, in the literature, there are no suitable cartridge sizes for thicker pancreas [8,44]. In the use of prophylactic drainage in DP/L, the literature has reported conflicting results: Ecker et al. observed that intraoperative drainage correlated with an increased POPF rate but decreased POPF intensity [28]. Miao et al., in the ISGPS consensus, stated, with a “weak” agreement, that standard abdominal drainage makes POPF and other postoperative complications, including collections after D/LP, more likely [8]. In the recent PANDORINA trial (an international, multicenter, open-label, randomized controlled, non-inferiority trial), the no-drain policy was observed to pose no increased risk regarding serious complications and led to fewer detections of grade B or C POPF [45]. The literature also reported conflicting results regarding the use of somatostatin analogues to reduce the rate of POPF after DP/L. Ecker et al. did not find a statistically significant association between the use of octreotide and CR-POPF. In contrast, two recent meta-analyses have found that somatostatin analogues are associated with less POPF after DP [46,47].

### 4.2. Limitations

This study has several limitations. (i) The results obtained from this meta-analysis are based on seven non-randomized studies that were assessed as having a “serious” overall bias by ROBINS-I, while the single RCT was assessed as having “some concerns” by RoB2. The high risk of bias among the individual studies included in this meta-analysis therefore requires caution in the analysis, interpretation of the results and their transferability in common clinical practice; (ii) the reliability of the pooled results from various indications, such as benign and malignant, along with a range of autologous and artificial coverage techniques, could be questioned. However, this issue has been partially addressed through subcategory analysis; (iii) only one of the included studies was a randomized controlled trial (RCT), while the others were all small retrospective comparative studies. All the studies come from Asian countries (Japan and South Korea), which limits the ability to generalize the results and apply them to Western countries. Western countries have different lifestyles and often treat patients with higher BMIs, different diseases, and varying therapeutic strategies, as well as different surgical skills; (iv) already extensively highlighted, when interpreting the results, it is essential to consider the significant variability of confounding factors. These include the closure technique associated with the mesh wrapping technique, the technique used in the control group and the perioperative treatment. The latter encompasses the combination or absence of splenectomy, the thickness and consistency of the pancreas, and the use of drainage and somatostatin analogues; (v) all studies used the ISGPS guidelines as the definition and classification criteria for POPF, although some studies referred to the first version, while others used the second version from 2017. These variations, although they are limitations of this meta-analysis, also reflect the significant variability in current clinical practice. Given the high frequency of POPF after D/LP and its impact on both clinical outcomes and healthcare costs, we believe that determining the best technique for managing the pancreatic stump is an important research topic.

This meta-analysis highlighted the potential benefits of mesh wrapping techniques for the pancreatic stump, particularly in reducing both overall POPF and CR-POPF, as well as mitigating the postoperative impact of POPF. Furthermore, with the necessary precautions, particularly due to the presence of serious bias in the individual studies analyzed, we believe this approach can be implemented in common clinical practice (through its application in clinical trials) as a reduction in the rate of POPF has been observed and no additional risk has been found in wrapping the mesh around the pancreatic stump. However, before recommending the use of this technique, further randomized controlled trials are needed to outline these benefits for each subgroup and to determine which techniques and products should be used. It would be desirable for RCTs to be conducted on large populations, preferably in a multi-center setting, with clear stratification based on the mentioned POPF risk factors and minimizing the number of confounding factors (thus avoiding the combination of interventions).

## 5. Conclusions

This systematic review and meta-analysis complements and enhances the existing literature, providing a contemporary analysis that incorporates recent advancements in managing the pancreatic stump after DP/L, with the aim of achieving a lower rate of POPF. Wrapping the pancreatic stump with mesh is associated with a decrease in overall POPF and clinically relevant POPF, a decrease in estimated blood loss, a reduced period with the surgical drain, and a decreased length of hospital stay. Despite the limitations mentioned above, the technique of wrapping the mesh around the pancreatic stump after D/LP appears to be a safe and feasible option. Currently, POPF is a highly significant issue in the surgical field, and there are still gaps in our understanding of it. It is therefore essential to conduct high-quality methodological studies to identify the risk factors for the onset of POPF and to evaluate and compare the results of various surgical approaches used to reduce its rate and associated morbidity.

## Figures and Tables

**Table 1:** Study and population characteristics. RCS = retrospective cohort studies; RCT = randomized controlled trial; PGA = polyglycolic acid; MPD = Main Pancreatic Duct; RSSC = Reinforced Stapler Suture Closure; NRU = Not Routinely Used; n = number; N/A = Not available.

Authors/Year	Study Type	Study Period	Study Country	Type of Mesh	Cohort Sample	Study Groups	No of Patients [n]	Sex (m/f) [n]	Age [Years] Mean (SD)	BMI [kg/m^2^] Mean (SD)
**Baba** et al., 2025 [18]	RCS	2020 to 2023	Japan	PGA	148	PGA mesh + Reinforced stapler suture closure + fibrin glue	25	11/14	67.4 (8.9)	21.3 (4.5)
						Reinforced stapler suture closure	123	55/68	67.6 (13.6)	22.1 (2.9)
**Imamura** et al., 2024 [19]	RCS	Jan 2013 to Aug 2023	Japan	PGA	165	PGA mesh + powered stapler closure + fibrin glue	115	58/57	68.5 (11.6)	23.1 (3.8)
						Manual stapler closure or hand-sewn closure	50	23/27	67.1 (14.4)	21.7 (2.5)
**Jang** et al., 2017 [20]	RCT	Nov 2011 to Apr 2014	Korea	PGA	97	PGA + fibrin glue	44	19/25	59.9 (12)	N/A
						Stapler closure	53	20/33	54.5 (14.1)	N/A
**Kawasaki** et al., 2022 [21]	RCS	Jan 2018 to Jun 2021	Japan	PGA	127	PGA + fibrin glue	17	N/A	N/A	N/A
						Reinforced stapler suture closure or stapler closure or MPD ligation	110	N/A	N/A	N/A
**Murata** et al., 2025 [22]	RCS	Feb 2011 to Jul 2024	Japan	PGA	131	PGA + transpancreatic mattress suture	18	11/7	63.1 (23.3)	22.5 (3.5)
						Hand-sewn closure (fish–mouth manner)	22	13/9	58.0 (20.6)	22.4 (3.1)
						Reinforced stapler suture closure	91	43/48	66.2 (19.6)	22.1 (3.2)
**Ochiai** et al., 2010 [23]	RCS	May 2003 to Apr 2008	Japan	PGA	63	PGA mesh + fibrin glue + Reinforced stapler suture closure (NRU)	26	N/A	N/A	N/A
						Hand-sewn or stapler closure	37	N/A	N/A	N/A
**Yoshida** et al., 2019 [24]	RCS	Jan 2010 to May 2018	Japan	Polyglactin	165	Polyglactin mesh + transpancreatic mattress suture	71	44/27	64.9 (11.8)	21.4 (3.7)
						Hand-sewn closure	94	48/46	67.1 (13.5)	22.3 (4.6)
**Yoshino** et al., 2019 [25]	RCS	Jan 2006 to Dec 2017	Japan	PGA	146	PGA mesh + fibrin glue	114	N/A	N/A	N/A
						Hand-sewn or stapler closure	32	N/A	N/A	N/A

**Table 2:** Post-Operative Pancreatic Fistula characteristics. PGA = polyglycolic acid; RSSC = Reinforced Stapler Suture Closure; MPD = Main Pancreatic Duct; NRU = Not Routinely Used; POPF = Post-Operative Pancreatic Fistula; Ov = Overall; BL = Biochemical Leak; n = number; N/A = Not available.

Authors/Year	Study Groups	No of Patients [n]	Overall POPF [n]	BL/Grade A POPF [n]	Grade B POPF [n]	Grade C POPF [n]	CR-POPF [n]
**Baba** et al., 2025 [18]	PGA mesh + Reinforced stapler suture closure + fibrin glue	25	11	6	N/A	N/A	5
	Reinforced stapler suture closure	123	78	24	N/A	N/A	54
**Imamura** et al., 2024 [19]	PGA mesh + powered stapler closure + fibrin glue	115	56	32	24	0	24
	Manual stapler closure or hand-sewn closure	50	25	5	20	0	20
**Jang** et al., 2017 [20]	PGA + fibrin glue	44	29	24	5	0	5
	Stapler closure	53	29	14	14	1	15
**Kawasaki** et al., 2022 [21]	PGA + fibrin glue	17	7	3	N/A	N/A	4
	Reinforced stapler suture closure or stapler closure or MPD ligation	110	75	23	N/A	N/A	52
**Murata** et al., 2025 [22]	PGA + transpancreatic mattress suture	18	7	6	1	0	1
	Hand-sewn closure (fish–mouth manner)	22	11	5	5	1	6
	Reinforced stapler suture closure	91	41	28	13	0	13
**Ochiai** et al., 2010 [23]	PGA mesh + fibrin glue + Reinforced stapler suture closure (NRU)	26	11	10	N/A	N/A	1
	Hand-sewn or stapler closure	37	21	11	N/A	N/A	10
**Yoshida** et al., 2019 [24]	Polyglactin mesh + transpancreatic mattress suture	71	49	45	4	0	4
	Hand-sewn closure	94	82	66	15	1	16
**Yoshino** et al., 2019 [25]	PGA mesh + fibrin glue	114	15	N/A	N/A	N/A	N/A
	Hand-sewn or stapler closure	32	11	N/A	N/A	N/A	N/A

**Table 3:** Intra-operative and post-operative outcomes characteristics. EBL = Estimated blood loss; PGA = polyglycolic acid; RSSC = Reinforced Stapler Suture Closure; MPD = Main Pancreatic Duct; NRU = Not Routinely Used; CD = Clavien-Dindo; n = number; N/A = Not available.

Authors/Year	Study Groups	No of Patients [n]	Operative Time [min] Mean (SD)	Estimated Blood Loss [ml] Mean (SD)	Time Required to Drain Removal [Days] Mean (SD)	Post-op. Complication (CD ≥ III) [n]	Length of Hospital Stay [Days] Mean (SD)	No of Re-Admission [n]
**Baba** et al., 2025 [18]	PGA mesh + Reinforced stapler suture closure + fibrin glue	25	348.5 (135.4)	68.1 (100.5)	6.5 (7.4)	2	11.2 (4.3)	1
	Reinforced stapler suture closure	123	347.5 (74.7)	98.6 (121.3)	11.8 (28.4)	11	14.3 (7.0)	4
**Imamura** et al., 2024 [19]	PGA mesh + powered stapler closure + fibrin glue	115	347.7 (25.3)	58.4 (27.5)	N/A	25	16 (6.8)	3
	Manual stapler closure or hand-sewn closure	50	338.5 (27.9)	109.3 (39.7)	N/A	45	22.3 (13)	1
**Jang** et al., 2017 [20]	PGA + fibrin glue	44	152.6 (58.3)	175 (175.9)	N/A	N/A	9.9 (7.5)	N/A
	Stapler closure	53	157.9 (57.6)	206.8 (166.3)	N/A	N/A	10 (6.3)	N/A
**Kawasaki** et al., 2022 [21]	PGA + fibrin glue	17	N/A	N/A	9.6 (6.9)	N/A	13.6 (7.7)	N/A
	Reinforced stapler suture closure or stapler closure or MPD ligation	110	N/A	N/A	23.4 (21)	N/A	27.7 (19.2)	N/A
**Murata** et al., 2025 [22]	PGA + transpancreatic mattress suture	18	501.9 (118.6)	130.1 (183.4)	N/A	2	12 (3.2)	1
	Hand-sewn closure (fish–mouth manner)	22	372.2 (242.5)	223 (450.1)	N/A	3	14 (4.8)	1
	Reinforced stapler suture closure	91	352.8 (95.7)	106.5 (163.5)	N/A	15	12.4 (4.5)	9
**Ochiai** et al., 2010 [23]	PGA mesh + fibrin glue + Reinforced stapler suture closure (NRU)	26	N/A	N/A	N/A	N/A	N/A	N/A
	Hand-sewn or stapler closure	37	N/A	N/A	N/A	N/A	N/A	N/A
**Yoshida** et al., 2019 [24]	Polyglactin mesh + transpancreatic mattress suture	71	232.5 (96.8)	1107.7 (1787.9)	N/A	9	14.5 (12.2)	4
	Hand-sewn closure	94	205.9 (86.2)	781.6 (879.4)	N/A	20	22.8 (39.2)	7
**Yoshino** et al., 2019 [25]	PGA mesh + fibrin glue	114	N/A	N/A	N/A	N/A	N/A	N/A
	Hand-sewn or stapler closure	32	N/A	N/A	N/A	N/A	N/A	N/A

## Data Availability

The data presented in this study are available on request from the corresponding author.

## Supplementary Materials

The following supporting information can be downloaded at: https://www.mdpi.com/article/10.3390/medicina61091688/s1, Figure S1: Estimated Blood Loss leave-one-out method; Table S1: Surgical and histopathological characteristics; File S1. Search Strategy; File S2. GRADE Summary of Findings 1.

## Abbreviations

The following abbreviations are used in this manuscript:

PRISMA	Preferred Reporting Items for Systematic Reviews and Meta-Analyses
SoF	summary of findings
RCS	retrospective cohort studies
RCT	randomized controlled trial
PGA	polyglycolic acid
MPD	Main Pancreatic Duct
RSSC	Reinforced Stapler Suture Closure
NRU	Not Routinely Used
n	number
N/A	Not available
POPF	Post-Operative Pancreatic Fistula
BL	Biochemical Leak
CD	Clavien-Dindo
CR	Clinically Relevant
RevMan	Review Manager
OR	odds ratios
Cis	confidence intervals
MH	Mantel-Haenszel
WMD	weighted mean differences
SD	standard deviation
IQR	interquartile range
OP	operative time
WMG	Wrapping Mesh Group
CG	Control group
