# Supplementary material for: Impact of Pancreatic Stump Wrapping with Mesh on Post-Operative Pancreatic Fistula in Patients Undergoing Distal/Left Pancreatectomy for Malignant or Benign Diseases: A Systematic Review and Meta-Analysis
Source: Medicina (Kaunas). 2025 Sep 17;61(9):1688. doi: 10.3390/medicina61091688 (PMC12472175; doi:10.3390/medicina61091688)
Supplement: Supplementary file 1 [file medicina-61-01688-s001.zip › Supplementary Table S1.pdf]

Supplementary Table S1. Surgical and histopathological characteristics.

Authors/year	Group	D/LP approach	Spleen-Preserving<br>*n*	Surgical drain placement	Indication PDAC/Total
**Baba** et al. / 2025	PGA mesh+ RSSC + fibrin glue	14 Lap<br>11 Rob	2	Yes	84/148
	RSSC	101 Lap<br>22 Rob	5		
**Imamura** et al. / 2024	PGA mesh + powered stapler closure + fibrin glue	67 Open<br>48 MIS	6	Yes	87/165
	Manual stapler closure or hand-sewn closure	40 Open<br>10 MIS	2		
**Jang** et al. / 2017	PGA + fibrin glue	Na	9	Yes	28/97
	Stapler closure		11		
**Kawasaki** et al. / 2022	PGA + fibrin glue	65 Open<br>61 Lap<br>1 Rob	Na	Yes	92/127
	RSSC or stapler closure or MPD ligation				
**Murata** et al. / 2025	PGA + transpancreatic mattress suture	18 Rob<br>0 Lap	13	Yes	10/18
	Hand-sewn closure (fish – mouth manner)	0 Rob<br>22 Lap	14		1/22
	RSSC	75 Rob<br>16 Lap	69		31/91
**Ochiai** et al. / 2010	PGA mesh + fibrin glue + RSSC (NRU)	Na	Na	Yes	Na
	Hand-sewn or stapler closure				
**Yoshida** et al. / 2019	Polyglactin mesh + transpancreatic mattress suture	165 Open	Na	Yes	39/71
	Hand-sewn closure				34/94
**Yoshino** et al. / 2019	PGA mesh + fibrin glue	57 Lap<br>89 Open	Na	Yes	Na
	Hand-sewn or stapler closure				

PGA = polyglycolic acid; MPD = Main Pancreatic Duct; RSSC = Reinforced Stapler Suture Closure; NRU = Not Routinely Used; n = number; Na = Not available; PDAC = Pancreatic ductal adenocarcinoma; Lap = Laparoscopic; Rob = Robotic; MIS = Minimally invasive surgery
