# Supplementary material for: Impact of Pancreatic Stump Wrapping with Mesh on Post-Operative Pancreatic Fistula in Patients Undergoing Distal/Left Pancreatectomy for Malignant or Benign Diseases: A Systematic Review and Meta-Analysis
Source: Medicina (Kaunas). 2025 Sep 17;61(9):1688. doi: 10.3390/medicina61091688 (PMC12472175; doi:10.3390/medicina61091688)
Supplement: Supplementary file 1 [file medicina-61-01688-s001.zip › File S2. GRADE Summary of Findings 1.pdf]

Summary of Findings 1. Wrap the mesh around the pancreatic stump (mesh group) after distal/left pancreatectomy to reduce post-operative pancreatic fistula compared to standard treatments or alternative treatment (control group)

Outcome and follow-up	Patients (studies), N	Relative effect (95% CI)	Absolute effects (95% CI)			Certainty	What happens
			Control group	Mesh group	Difference		
**Overall post-operative pancreatic fistula**	1042 (8 studies)	**RR = 0.77** (0.60 to 0.95)	**Study population**			⊕⊕◯◯ Low[b,c]	Wrapping mesh around the pancreatic stump after distal/left pancreatectomy may decreases the number of overall post-operative pancreatic fistula
			609 per 1.000	**469 per 1.000** (366 to 579)	**140 fewer per 1.000** (from 244 fewer to 30 fewer)		
			**Low**				
			343 per 1.000[a]	**264 per 1.000** (206 to 326)	**79 fewer per 1.000** (from 137 fewer to 17 fewer)		
			**High**				
			872 per 1.000[a]	**671 per 1.000** (523 to 828)	**201 fewer per 1.000** (from 349 fewer to 44 fewer)		
**Clinically relevant post-operative pancreatic fistula**	896 (7 studies)	**RR = 0.42** (0.28 to 0.60)	**Study population**			⊕⊕⊕◯ Moderate[b]	Wrapping mesh around the pancreatic stump after distal/left pancreatectomy probably decreases the number of clinically relevant post-operative pancreatic fistula
			321 per 1.000	**135 per 1.000** (90 to 192)	**186 fewer per 1.000** (from 231 fewer to 128 fewer)		
			**Low**				
			170 per 1.000[a]	**71 per 1.000** (48 to 102)	**99 fewer per 1.000** (from 122 fewer to 68 fewer)		
			**High**				
			472 per 1.000[a]	**198 per 1.000** (132 to 283)	**274 fewer per 1.000** (from 340 fewer to 189 fewer)		

CI: confidence interval; **RR:** risk ratio

GRADE Working Group grades of evidence

High certainty: we are very confident that the true effect lies close to that of the estimate of the effect.

Moderate certainty: we are moderately confident in the effect estimate: the true effect is likely to be close to the estimate of the effect, but there is a possibility that it is substantially different.

Low certainty: our confidence in the effect estimate is limited: the true effect may be substantially different from the estimate of the effect.

Very low certainty: we have very little confidence in the effect estimate: the true effect is likely to be substantially different from the estimate of effect.

a. The low and high-risk values are respectively the lowest and highest rate of post-operative pancreatic fistulas in the studies analyzed in this review

b. We downgraded by one level due to the serious risk of bias in the D1 domain (bias due to confounding) present in all observational studies included in this review

c. We downgraded one level for serious inconsistency due to substantial statistical heterogeneity with an I2 of 50%, with a p-value of 0.05.
