# Supplementary material for: Impact of Pancreatic Stump Wrapping with Mesh on Post-Operative Pancreatic Fistula in Patients Undergoing Distal/Left Pancreatectomy for Malignant or Benign Diseases: A Systematic Review and Meta-Analysis
Source: Medicina (Kaunas). 2025 Sep 17;61(9):1688. doi: 10.3390/medicina61091688 (PMC12472175; doi:10.3390/medicina61091688)
Supplement: Supplementary file 1 [file medicina-61-01688-s001.zip › File S1.Search Strategy.pdf]

- PubMed/MEDLINE

((pancreas[MeSH terms] OR pancreas[tiab] OR pancreatic[tiab] OR pancreato*[tiab]) AND (resection* [tiab] OR surger* [tiab] OR surgical [tiab])) OR left pancreatectom*[tiab] OR distal pancreatectom*[tiab] OR DP[tiab] OR pancreatectom*[tiab] OR distal pancreatectomy[Mesh] OR pancreatectomy[MeSH] AND polyglycolic acid mesh*[tiab] OR surgical mesh*[tiab] OR mesh*[tiab] OR felt*[tiab] OR wrap*[tiab] OR fibrin[tiab]

- Web of Science

(TI=(((pancreas OR pancreas OR pancreatic OR pancreato*) AND (resection* OR surger* OR surgical)) OR (left pancreatectom* OR distal pancreatectom* OR pancreatectom*))) AND TS=((polyglycolic acid mesh* OR surgical mesh* OR mesh* OR felt* OR wrap* OR fibrin))

- Scopus

[Article title, abstract, Keywords] ((pancreas OR pancreas OR pancreatic OR pancreato*) AND (resection* OR surger* OR surgical)) OR (left AND pancreatectom* OR distal AND pancreatectom* OR pancreatectom*) AND [Article title, abstract, Keywords] (polyglycolic AND acid AND mesh* OR surgical AND mesh* OR mesh* OR felt* OR wrap* OR fibrin)
