# Supplementary figures and images for: Impact of Pancreatic Stump Wrapping with Mesh on Post-Operative Pancreatic Fistula in Patients Undergoing Distal/Left Pancreatectomy for Malignant or Benign Diseases: A Systematic Review and Meta-Analysis
Source: Medicina (Kaunas). 2025 Sep 17;61(9):1688. doi: 10.3390/medicina61091688 (PMC12472175; doi:10.3390/medicina61091688)

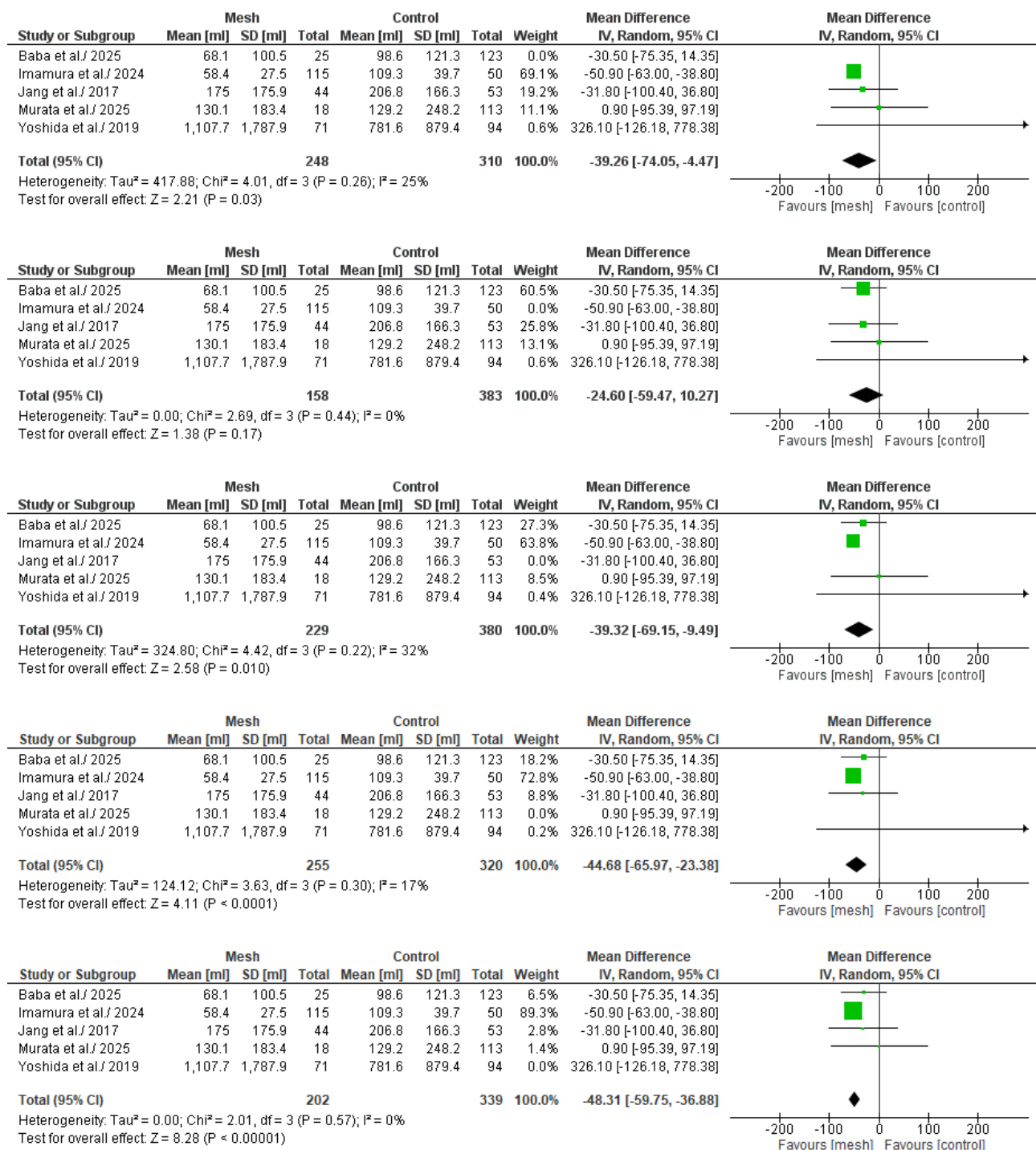

**Figure S1: Estimated Blood Loss leave-one-out method**

Supplement: Supplementary file 1 [file medicina-61-01688-s001.zip › Figure S1 Estimated Blood Loss leave-one-out method.pdf]
